# Analysis of neurosensory adverse events induced by FOLFOX4 treatment in colorectal cancer patients: a comparison between two Asian studies and four Western studies

**DOI:** 10.1002/cam4.25

**Published:** 2012-08-06

**Authors:** Kenichi Sugihara, Atsushi Ohtsu, Yasuhiro Shimada, Nobuyuki Mizunuma, Katsushige Gomi, Po-Huang Lee, Aimery Gramont, Mace L Rothenberg, Thierry André, Silvano Brienza, Richard M Goldberg

**Affiliations:** 1 Department of Surgical Oncology, Tokyo Medical and Dental UniversityTokyo, Japan; 2 Research Center for Innovative Oncology, National Cancer Center Hospital EastKashiwa, Japan; 3 Gastrointestinal Oncology, National Cancer Center HospitalTokyo, Japan; 4 Medical Oncology, Cancer Institute HospitalTokyo, Japan; 5 Yakult Honsha Co., Ltd.Tokyo, Japan; 6 Department of Surgery, National Taiwan University HospitalTaipei, Taiwan; 7 Department of Medical Oncology, Hospital Saint-AntoineParis, France; 8 Phase I Drug Development, Vanderbilt-Ingram Cancer CenterNashville, Tennessee; 9 Debiopharm S.A.Lausanne, Switzerland; 10 Comprehensive Cancer Center, Ohio State UniversityColumbus, Ohio

**Keywords:** Colorectal cancer, ethnic difference, FOLFOX4, neurotoxicity, oxaliplatin

## Abstract

The grades of neurosensory adverse events (NSAEs) induced by FOLFOX4 treatment were compared between Asian and Western colorectal cancer patients and correlated with cumulative oxaliplatin doses. A total of 3359 patients treated with FOLFOX4 were analyzed: 1515 from two Asian studies (Japanese Post Marketing Surveillance [J-PMS] and MASCOT) and 1844 from four Western studies (EFC2962, N9741, EFC4584, and MOSAIC). The onset of NSAEs was analyzed in terms of treatment duration and cumulative dose of oxaliplatin. The incidence of grade ≥3 NSAEs ranged from 2.0% to 4.4% in Asian studies and 9.3% to 19% in Western studies. The cumulative doses of oxaliplatin that induced grade ≥3 NSAEs in 10% of patients were higher in Asian studies (1526 mg/m^2^ or not reached) than in Western studies (805–832 mg/m^2^). No significant correlations were noted between occurrence of grade ≥3 NSAEs and demographic/baseline characteristics. The frequency of escalation from grade 0 to 1 in J-PMS was statistically significantly lower than that in EFC4584, and that from grade 0 to 1 and from grade 1 to 2 in MASCOT lower than that in MOSAIC. The cumulative oxaliplatin doses administered during grade escalation in J-PMS were similar to those in EFC2962 or EFC4584. All grade-3 NSAEs in MASCOT and 96% of those in MOSAIC improved to grade 2 or less within 12 months of follow-up. The Asian populations accrued to these studies appear to be less susceptible to the neurotoxicity of oxaliplatin than the mainly Caucasian populations in the Western studies.

## Introduction

FOLFOX4, a combination regimen of 5-fluorouracil (5-FU), leucovorin (LV), and oxaliplatin, was established as a standard regimen for treatment of metastatic colorectal cancer in the first- and second-line settings in Western countries [1–4]. Similar efficacy of the FOLFOX4 regimen was reported in Asian and Japanese patients, although the studies included fewer patients [5–8]. To evaluate the safety of FOLFOX regimens in Japanese patients with metastatic colorectal cancer, a study termed “Post Marketing Surveillance” (J-PMS) was conducted in Japan. FOLFOX4 is also a standard-adjuvant treatment for Stage III colon cancer in Western and Asian countries based on the results of the MOSAIC and the MASCOT studies [9–11]. Since then, the FOLFOX4 regimen has been used in the pivotal regulatory studies as a control arm [12–15]. It has also contributed to the establishment of other FOLFOX regimens [16].

‘Oxaliplatin has a characteristic side-effect profile that includes neurotoxicity and hypersensitivity, and management requires appropriate monitoring and potential adjustment of treatment [17–20]. Oxaliplatin-induced neurotoxicity is classified as either acute or chronic based on the onset time after treatment [17, 18]. Oxaliplatin appears to affect neuronal voltage-gated sodium channels in both acute and chronic neuropathy [21, 22], while damage of dorsal root ganglia is reported as the cause of chronic neurotoxicity [23, 24]. Infusions of calcium and magnesium have been used in an attempt to prevent neurotoxicity with some reports showing a decrease in severity, but this intervention does not appear to be preventive [17, 18, 25, 26].

Recently, attention has been drawn to racial- or ethnicity-related differences in both the prevalence of colorectal cancer and the therapeutic response to chemotherapy [27, 28]. We previously reported the results of a comparative safety analysis of six FOLFOX4-regulatory studies with a total of 3359 colorectal cancer patients: 1515 from two Asian studies (J-PMS and MASCOT [11]) and 1844 from four Western studies (EFC2962 [1], N9741 [2], EFC4584 [3, 4], and MOSAIC [9, 10]) [29]. This safety comparison had been performed to prepare the supplemental new-drug application (NDA) documents to obtain the regulatory approval of the FOLFOX regimen as a colon cancer-adjuvant indication in Japan. Considering the regulatory nature of this objective, the four pivotal Western studies, which included FOLFOX4 arm, were selected. J-PMS was a postmarketing surveillance and its design was thus different from the other five studies. Treatment regimens used were not limited to FOLFOX4, but only those patients who were to receive a standard FOLFOX4 regimen were extracted in our analysis. As a result, the dose intensity in the population used in this analysis was similar to that in the other five studies. This result, together with the demographic and baseline characteristics, suggests that the comparison of safety data between the two Asian and the four Western studies was feasible and informative. There was no evidence that Asian patients experienced worse toxicity than did Western patients. Unexpectedly, the probability of grade ≥3 neurosensory adverse events (NSAEs) presented by the Kaplan–Meier curve in J-PMS and MASCOT was statistically significantly lower than that in EFC4584 and MOSAIC, respectively.

Here, we further investigated the NSAEs induced by FOLFOX4 treatment in terms of worsening toxicity grade and cumulative oxaliplatin dose in the same six studies.

## Materials and Methods

### Studies analyzed

Safety data of patients treated with FOLFOX4 were extracted from the same six studies, of which profiles were described in our previous article [29] and Table 1. These studies were classified to either Asian or Western based on the study countries. The racial characteristics showed that all patients were oriental/Asian in two Asian studies. One exception was MOSAIC study, in which Australia, Israel, and Singapore joined the study in addition to many European countries. The other exception was the EFC2962 study, in which Israel joined the study in addition to many European countries. However, as the number of oriental/Asian patients included in four Western studies was very small (0.5–2%), their influence on the objective of this analysis (comparison of ethnic differences) was deemed negligible. J-PMS was a prospective survey conducted in Japanese patients with metastatic colorectal cancer, who were receiving FOLFOX including FOLFOX4. It was conducted to comply with a conditional approval commitment given by the Japanese regulatory authority. As no pivotal FOLFOX studies had been conducted in Japan at that time, J-PMS was included in this safety analysis. Among 5119 patients enrolled between April 2005 and March 2006, the experiences of 1356 patients treated with the standard FOLFOX4 regimen were extracted. Among 1356 patients, 222 (16%) patients were previously untreated with chemotherapy and 1134 (84%) patients were previously treated with chemotherapy. FOLFOX4 regimen was used as the second line in 430 (32%) patients, and as the third-line or later treatment in 701 (52%) patients; not recorded in three patients. Four pivotal Western studies were selected because these studies had been conducted to obtain the regulatory approval of FOLFOX4 for the indications for first- or second-line treatment of metastatic disease and for adjuvant treatment for disease after resection [1–4, 9, 10]. Furthermore, the data from the MASCOT study in Asian populations [11] were compared with those in the MOSAIC study conducted in Western populations [9, 10]. Based on the results of the MOSAIC study, FOLFOX4 became a standard-adjuvant treatment of colon cancer in Western countries. MASCOT study was conducted in five Asian countries to evaluate the safety and tolerability of adjuvant FOLFOX4 in Asian patients. Its study design was almost identical to that of FOLFOX4 arm in the MOSAIC study. Therefore, the comparison of MASCOT with FOLFOX4 arm of MOSAIC seemed to be rational and appropriate.

**Table 1:** Cumulative dose of oxaliplatin linked to neurosensory adverse events

	Asian studies		Western studies			
		
Parameters	J-PMS	MASCOT	EFC2962	N9741	EFC4584	MOSAIC
Phase	PMS	IV	III	III	III	III
Treatment	Any lines	Adjuvant	First	First	Second	Adjuvant
Patients	Metastatic	Stage II/III	Metastatic	Metastatic	Metastatic	Stage II/III
Patients treated with FOLFOX4	1356	159	209	259	268	1108
Grade ≥1						
Patient no. (%)	704 (52)	133 (84)	173 (83)	207 (80)	201 (75)	1020 (92)
CD_10_ (mg/m^2^)	85	85	85	85	85	85
Grade ≥2						
Patient no. (%)	297 (22)	37 (23)	124 (59)	107 (41)	60 (22)	487 (44)
CD_10_ (mg/m^2^)	405	782	255	292	505	337
Grade ≥3						
Patient no. (%)	27 (2.0)	7 (4.4)	39 (19)	47 (18)	25 (9.3)	137 (12)
CD_10_ (mg/m^2^)	1526	NR	805	827	821	832

The cumulative oxaliplatin doses that induced grade ≥1, 2, or 3 neurosensory adverse events in 10% of patients (CD_10_) were calculated by Kaplan–Meier method.

NR, not reached; PMS, postmarketing surveillance.

The FOLFOX4 regimen is oxaliplatin 85 mg/m^2^ on day 1, LV 200 mg/m^2^ per day or *l*-LV 100 mg/m^2^ per day on days 1 and 2, and 5-FU bolus 400 mg/m^2^ per day followed by continuous infusion 600 mg/m^2^ per day on days 1 and 2, repeated every 2 weeks. For patients to be eligible for this analysis in J-PMS, they must have received oxaliplatin 80–90 mg/m^2^, *l*-LV 75–125 mg/m^2^ per day, and 5-FU bolus 350–450 mg/m^2^ per day followed by continuous infusion 550–650 mg/m^2^ per day on the same schedule at least in the first cycle of therapy.

### Safety analysis

All data were collected prospectively in the six studies, provided in SAS® version 8.1 format (SAS Institute, Cary, NC), and analyzed by using the same SAS programs for the six studies. As a result, the data obtained were appropriately normalized to permit an accurate interstudy comparison of the results. Descriptive analyses were performed to evaluate dose duration and cumulative dose, and percentages, means and standard deviations were provided. As peripheral neurotoxicity was defined in the six studies, respectively, to standardize its definition, it was named as NSAE and those recorded in the database of each study were recoded and grouped under the common MedDRA (the Medical Dictionary for Regulatory Activities) Preferred Terms (version 9.0). These Preferred Terms included neuropathy peripheral, paresthesia, dysesthesia, peripheral sensory neuropathy, sensory disturbance, hypoesthesia, hypoesthesia facial, hypoesthesia oral, neuropathy, neurotoxicity, and neurological symptom. They were graded by the Neurotoxicity Criteria of Debiopharm, Lausanne, Switzerland (DEB-NTC) [7] in J-PMS or by the National Cancer Institute Common Toxicity Criteria (NCI-CTC) version 1 in EFC2962 and MOSAIC or version 2 in MASCOT, N9741, and EFC4584.

The incidence of grade 1–4 NSAEs was calculated across all treatment cycles in all six studies, and the frequency from a lower to a higher grade was analyzed. If more than one NSAE of the same grade was observed in the same patient, the first observed one was used for the analysis of progression to a higher grade of toxicity. If more than one grade was documented on the same day, the higher grade was used. If the information on the date of onset was missing or incomplete, then onset at the first day of the cycle was assumed. If the information on onset date and cycle was missing or incomplete, these patients were excluded from the analysis of grade escalation. In J-PMS, EFC2962, and EFC4584 studies, the onset dates of NSAEs were available as well as the dates and doses of oxaliplatin administered so that the treatment duration and the cumulative doses of oxaliplatin could be analyzed. As the onset date of NSAEs was not recorded in MASCOT, N9741, and MOSAIC studies, only toxicity grade was analyzed.

The follow-up after study completion was defined in each individual study in routine bases, with special considerations for NSAEs in the adjuvant MASCOT and MOSAIC studies. The recovery of grade-3 NSAEs during the follow-up period of these two studies was analyzed and compared at 12 and 18 months, respectively. The patients whose grade of NSAE was not recorded were excluded from the analysis.

### Statistical analysis

The cumulative doses of oxaliplatin that induced grade ≥1, ≥2, or ≥3 NSAEs in 10% of patients (CD_10_) were calculated by the Kaplan–Meier method. The dose associated with toxicity in 10% of animals was considered to be important for the evaluation of safety [30] and we applied this parameter to our analysis. The correlation between occurrence of grade ≥3 NSAEs and demographic/baseline characteristics was analyzed by multivariate logistic regression. NR (not relevant) means that the number of patients in each category was insufficient to allow the interpretation of results, for example, an odds ratio >50. NA (not applicable) means the following: data were not collected for corresponding factors in the study, there was no grade ≥3 NSAE, or all patients were classified in the same category. The frequency of grade escalation of NSAEs was statistically compared by Fisher's exact test within the matched metastatic or adjuvant studies.

## Results

### Cumulative dose of oxaliplatin that induced NSAEs in 10% of patients

The CD_10_ values of grade ≥1 NSAEs were approximately 85 mg/m^2^ in all six studies; those of grade ≥2 NSAEs in two Asian studies (405–782 mg/m^2^) showed a higher trend than those in four Western studies (255–505 mg/m^2^); those of grade ≥3 NSAEs in two Asian studies (1526 mg/m^2^ or not reached) were clearly higher than those in four Western studies (805–832 mg/m^2^) (Table 1). In the MASCOT and MOSAIC studies, the numbers of patients who could receive 936–1020 mg/m^2^ of cumulative oxaliplatin doses were 101/159 (64%) and 403/1108 (36%), respectively (data not shown in Table 1). The incidences of grade ≥3 NSAEs were in the range of 2.0–4.4% in Asian studies and 9.3–19% in Western studies. In the original article of MASCOT study [11], grade ≥3 NSAEs were described in nine patients. Among them, two cases were observed during follow-up period, but not the study period, and these were excluded in this analysis. Grade-4 NSAEs were not observed in any studies.

### Multivariate analysis of grade ≥3 NSAEs

To investigate the risk factors of grade ≥3 NSAEs, the correlation between their occurrence and demographic/baseline characteristics was analyzed in the six studies by multivariate logistic regression (Table 2). There were no significant trends observed in any studies. Statistical significance was only observed in the hemoglobin values of MASCOT study.

**Table 2:** Multivariate analysis of occurrence of grade ≥3 neurosensory adverse events by odds ratio estimate

		Asian studies		Western studies			
			
Parameters		J-PMS	MASCOT	EFC2962	N9741	EFC4584	MOSAIC
Patient no.		1356	159	209	259	268	1108
Age							
<65 versus ≥65 years	Estimate	0.6161	2.4540	1.4393	1.2042	1.4760	0.9460
	95% CI	0.278–1.366	0.258–23.340	0.668–3.103	0.601–2.412	0.587–3.714	0.652–1.373
	*P*-value	0.2333	0.4347	0.3527	0.6000	0.4083	0.7700
Gender							
Female versus Male	Estimate	0.8912	2.3668	0.8182	1.3785	0.9546	1.1147
	95% CI	0.378–2.101	0.416–13.464	0.388–1.723	0.694–2.737	0.408–2.232	0.778–1.597
	*P*-value	0.7923	0.3314	0.5974	0.3590	0.9147	0.5540
Performance status							
≤1 versus ≥2	Estimate	1.4657	NR	1.9841	0.7881	NR	NR
	95% CI	0.191–11.264	NR	0.432–9.115	0.206–3.010	NR	NR
	*P*-value	0.7133	NR	0.3784	0.7277	NR	NR
Previous chemotherapy							
No versus Yes	Estimate	1.3728	NA	NA	NA	NR	NA
	95% CI	0.499–3.780	NA	NA	NA	NR	NA
	*P*-value	0.5398	NA	NA	NA	NR	NA
Previous surgery							
No versus Yes	Estimate	0.6919	NA	1.1534	0.4420	0.4177	NA
	95% CI	0.156–3.060	NA	0.393–3.381	0.173–1.128	0.053–3.282	NA
	*P*-value	0.6273	NA	0.7948	0.0876	0.4065	NA
Neutrophil count							
≥2000 versus <2000/mm^3^	Estimate	1.6380	NR	NR	NA	NR	NA
	95% CI	0.217–12.366	NR	NR	NA	NR	NA
	*P*-value	0.6323	NR	NR	NA	NR	NA
Hemoglobin							
≥10 versus <10 g/dL	Estimate	0.8806	0.1033	4.7763	1.4488	2.2013	NA
	95% CI	0.294–2.639	0.019–0.559	0.614–37.145	0.304–6.913	0.279–17.347	NA
	*P*-value	0.8204	0.0084	0.1351	0.6420	0.4538	NA

All data of platelet count (≥100,000 versus <100,000/mm^3^) are either NA or NR and not included.

CI, confidence interval; NA, not applicable; NR, not relevant.

### Frequency of grade escalation in NSAEs

We evaluated grade escalation first in the metastatic colorectal cancer studies (Table 3). In J-PMS, 45% of patients experienced grade 1, whereas among patients in EFC2962, N9741, and EFC4584 this occurred in 64–74%. The patients, whose NSAEs went from grade 0 to 2 were 6.6% in J-PMS and 3.0–13% in the other studies; those from grade 1 to 2 were 15% in J-PMS and 14–35% in the other studies. The number of patients, whose grade-0 NSAE escalated to grade 3 directly was very small in all four studies. The patients whose NSAEs escalated from grade 1 to 3 were 0.5% in J-PMS and 4.9–5.3% in the other studies; those from grade 2 to 3 were 1.3% in J-PMS and 4.1–13% in the other studies. The frequencies of escalations from grade 0 to 1, grade 0 to 2, grade 1 to 3, and grade 2 to 3 in J-PMS (84% of patients were previously treated with chemotherapy) were statistically significantly lower than those in EFC4584 (second-line treatment). Among all patients who experienced grade-2 NSAEs, those who withdrew shortly thereafter from the studies due to any reasons were 54/288 (19%), 6/101 (5.9%), 13/93 (14%), and 11/46 (24%) in J-PMS, EFC2962, N9741, and EFC4584 studies, respectively (data not shown in Table 3).

**Table 3:** Grade escalation of neurosensory adverse events

	Asian studies		Western studies			
		
Parameters	J-PMS	MASCOT	EFC2962	N9741	EFC4584	MOSAIC
Patient no.	1356	159	209	259	268	1108
Escalated patients (%)						
Grade 0–1	611 (45)	128 (81)	134 (64)^a^	192 (74)^a^	185 (69)^a^	980 (88)^b^
Grade 0–2 directly	90 (6.6)	5 (3.1)	27 (13)^a^	14 (5.4)	8 (3.0)^a^	36 (3.2)
Grade 1–2	198 (15)	30 (19)	74 (35)^a^	79 (31)^a^	38 (14)	383 (35)^b^
Grade 0–3 directly	2 (0.1)	0	3 (1.4)^a^	1 (0.4)	1 (0.4)	4 (0.4)
Grade 1–3 directly	7 (0.5)	2 (1.3)	11 (5.3)^a^	13 (5.0)^a^	13 (4.9)^a^	64 (5.8)^b^
Grade 2–3	18 (1.3)	5 (3.1)	23 (11)^a^	33 (13)^a^	11 (4.1)^a^	69 (6.2)

*P* < 0.05 versus J-PMS (a) or MASCOT (b) by Fisher's exact test.

Second, grade escalation was evaluated in the adjuvant studies. In MASCOT and MOSAIC, 81% and 88% of patients experienced grade 1, respectively. The patients, whose NSAEs went from grade 0 to 2, were 3.1% and 3.2%; those from grade 1 to 2 were 19% and 35%, respectively. The number of patients, whose grade-0 NSAE escalated to grade 3 directly was also very small in both studies. The patients, whose NSAEs escalated from grade 1 to 3 were 1.3% and 5.8%; those from grade 2 to 3 were 3.1% and 6.2%, respectively. The frequencies of escalations from grade 0 to 1, grade 1 to 2, and grade 1 to 3 in MASCOT were statistically significantly lower than those in MOSAIC.

### Duration and cumulative dose of oxaliplatin administered during grade escalation

**Figure 1:**
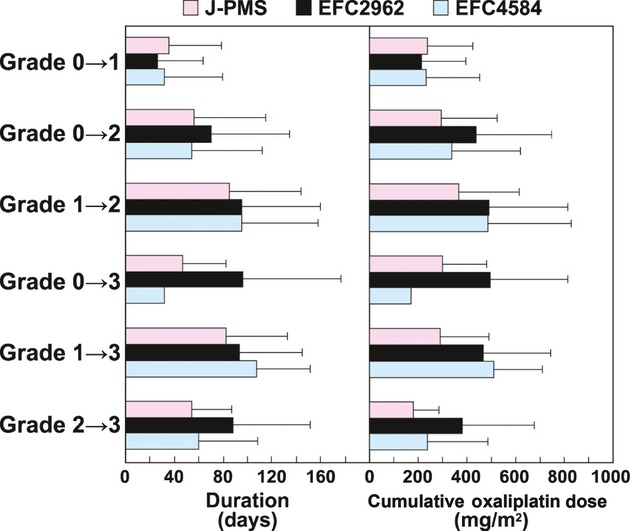
Treatment duration and cumulative dose of oxaliplatin administered during grade escalation of neurosensory adverse events. Mean and SD.

On the whole, the mean treatment duration and cumulative dose of oxaliplatin administered during each grade escalation in J-PMS were similar to those in EFC2962 and EFC4584 studies, including the escalation from grade 0 to 1 or 2 and from grade 1 to 2 (Fig. 1). Some difference was observed in the escalation from grade 2 to 3; namely, mean values of duration and cumulative dose in EFC2962 (88 days, 380 mg/m^2^) were more than those in J-PMS (54 days, 181 mg/m^2^) or EFC4584 (60 days, 236 mg/m^2^). As shown in Table 3, the number of patients, whose NSAEs escalated from grade 0 to 3 directly, was too small in these three studies to draw any conclusions.

**Figure 2:**
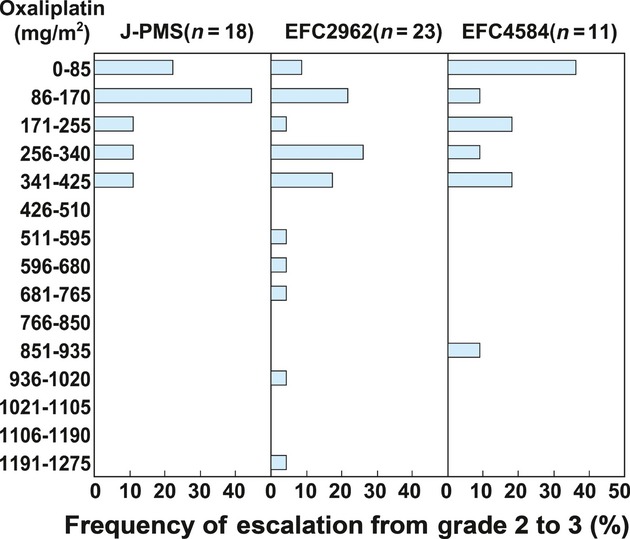
Cumulative dose of oxaliplatin administered during escalation of neurosensory adverse events from grade 2 to 3 in each patient.

The cumulative dose of oxaliplatin administered during the escalation from grade 2 to 3 was further analyzed in each patient (Fig. 2). All of 18 patients in J-PMS and 10 (91%) patients in EFC4584 experienced the grade escalation in the dose range from 0 to 425 mg/m^2^, while 18 (78%) patients in EFC2962 did. Five patients in EFC2962 experienced grade 3 in the dose range from 511 to 1275 mg/m^2^, whereas no patient in J-PMS and only one patient in EFC4584 did.

### Recovery of grade-3 NSAEs during follow-up period

**Figure 3:**
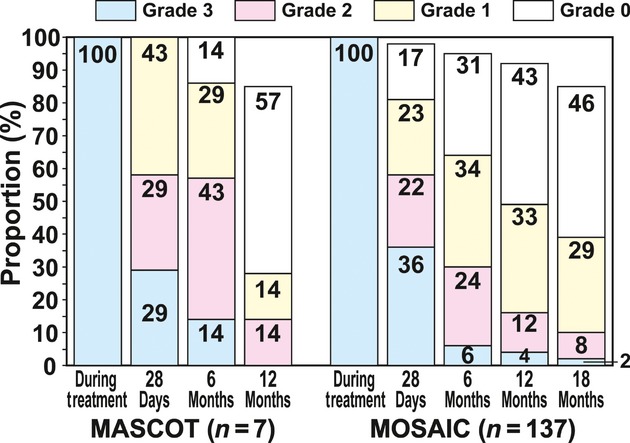
Recovery of grade-3 neurosensory adverse events during follow-up period.

The recovery of grade-3 NSAEs during the follow-up period was analyzed in the MASCOT and MOSAIC studies at 12 and 18 months, respectively (Fig. 3). In the follow-up at 12 months in MASCOT and at 28 days, 6, 12, and 18 months in MOSAIC, the grades of NSAEs were not recorded in 15, 2, 5, 8, and 15% of patients, respectively, and these patients were excluded from the analysis. In MASCOT, seven patients, who experienced grade-3 NSAEs during study period, recovered gradually, and at 12 months, all patients improved to grade 2 or less. In MOSAIC, 137 patients, who experienced grade-3 NSAEs, recovered gradually, and at 12 and 18 months, 4% and 2% of patients still continued to have grade 3, respectively. Seventy-one percent of patients in MASCOT and 75% of patients in MOSAIC improved to grade 1 or less during the follow-up period.

## Discussion

In our previous article, we reported the results of analyses of dose intensity, treatment duration, and cumulative oxaliplatin dose in the six studies and concluded that the comparison of safety data among these studies was feasible and informative [29]. This indicates that the analysis in this article is also informative.

This analysis confirms that the probability of the grade ≥3 NSAEs in Asian studies was less than that in Western studies as evidenced by the increase in cumulative oxaliplatin doses linked to the grade ≥3 NSAEs (Table 1). This finding is also supported by the analysis of grade escalation of NSAEs, which shows the lower frequencies in Asian studies than in Western studies in many cases (Table 3). We previously reported that 130 (82%) patients could complete 12 cycles of treatment in MASCOT, while 828 (75%) patients could do in MOSAIC (Fig. 1 in [29]). This difference was confirmed by the larger number of patients, who could receive 936–1020 mg/m^2^ of cumulative oxaliplatin doses, in MASCOT than in MOSAIC. This study also confirms the reports from the MOSAIC study that grade-3 NSAEs most often improved to grade 2 or less within 1 year of the follow-up in most of the patients (Fig. 3).

The lower incidence/probability of grade-3 NSEAs in Asian patients may be potentially explicable by either of the following two reasons:

- In Asian studies, after the first experience of grade-2 NSAEs, dose reduction/dose delay/discontinuation of oxaliplatin treatment was performed more aggressively, or more patients were withdrawn from the studies.
- The Asian populations were less susceptible to neurotoxicity of oxaliplatin than the Caucasian populations.

The former reason can be disregarded, because the mean values of treatment duration and cumulative dose of oxaliplatin administered were similar in all of J-PMS, EFC2962, and EFC4584 studies (Fig. 1). The difference between EFC2962 and J-PMS or EFC4584 observed in Figures 1 and 2 may be explained by that the patients in EFC2962, a first-line study, could receive longer FOLFOX4 treatment without disease progression. Early withdrawal of Asian patients from treatment can also be disregarded, because the number of patients withdrawn after the first experience of grade-2 NSAEs in J-PMS was not more than that in EFC4584. Thus, it is suggested that susceptibility to high-grade NSAEs may be less in Asian populations than in Caucasian populations.

Grade-3 NSAEs accrued only late in the course of oxaliplatin therapy and are different from acute NSAEs [17, 18]. Recent progress of pharmacogenetic analysis has elucidated the existence of genetic polymorphisms associated with delayed-type NSAEs caused by oxaliplatin treatment. The genetic polymorphisms of *GSTP1* gene, reported by Lecomte et al., were considered as a risk factor of delayed NSAE by oxaliplatin [31]. A homozygous variant genotype for *GSTP1* was also reported to be more commonly associated with the discontinuation of FOLFOX treatment due to neurotoxicity through a retrospective pharmacogenetic analysis of the N9741 study [32]. Another polymorphisms associating with NSAEs were reported in the AGT gene, which was involved in the metabolism of oxalate [33]. The ethnic difference in grade ≥3 NSAEs between Asian and Western patients observed in our analysis may be explained by the different frequencies of polymorphisms. Unfortunately, there have been no articles reported with respect to the ethnic difference between Asian and Western patients, including genetic polymorphisms. Such analysis was not possible because of lack of blood samples, on which pharmacogenetic analyses could be conducted, in most of the patients enrolled in these studies. Prospective investigations are expected in the future to elucidate these regards.

As we described previously, other possible explanations are that environmental and cultural factors such as patient's lifestyle, tolerance to or care for neurologic abnormality, and so forth, may have been relevant [29]. The care for NSAEs may have been more in patients of J-PMS and MASCOT. Further investigations are expected in these regards.

In this analysis, different versions of NSAE-grading systems were used, which may have given some impact on the results. The definition of grade-1 and -2 NSAEs included paresthesia in common, and dysesthesia, loss of deep tendon reflexes, or sensory loss, respectively; however, their persistency or severity described was not the same. This may have somewhat affected the incidences of these NSAEs in each study. Concerning grade-3 NSAEs, “the functional impairment/interfering with activities of daily living” were actually included in the definition of all grading criteria. Such analogous criteria made their comparison more appropriate, and resulted in the clearer conclusions.

Various risk factors have been reported concerning oxaliplatin-induced neurotoxicity, including treatment schedule, single dose per cycle, cumulative dose, infusion time, pre-existing peripheral neuropathy, and surgery [18]. Our results in Table 1 confirm that the cumulative dose is a critical risk factor of NSAEs. There were no other significant risk factors, including previous surgery, observed in the occurrence of grade ≥3 NSAEs in any studies (Table 2). The influences of treatment schedule, single dose per cycle, and infusion time were not analyzed as this safety analysis was focused on FOLFOX4 regimen in the six studies. Pre-existing NSAEs were also not analyzed as the patients with these symptoms were excluded from the enrollment in the five studies and those in J-PMS were less than 1.1% of total safety population (data not shown).

Prior chemotherapies and concomitant medications are also possible confounders of incidence of NSAEs. In all six studies generally, premedications for allergy and for nausea and vomiting, including 5-HT_3_ inhibitors and steroids, were allowed as well as supportive therapies such as drugs for pain management. As any influences of these medications or supportive therapies on the incidence of NSAEs have not been reported so far, the usages of these medications or supportive therapies deem to have given no significant impact on the results of our analysis.

Concerning chemotherapeutic agents, their prior usages were not allowed in the first-line studies (EFC2962 and N9741) and the adjuvant studies (MOSAIC and MASCOT). In EFC4584, only 5-FU/LV plus irinotecan, non-neurotoxic drugs, was allowed as a prior chemotherapy, which had to be completed at least 3 weeks prior to randomization. Although J-PMS was a postmarketing surveillance and prior chemotherapies were not restricted, major prior chemotherapies included 5-FU, its analogs, and irinotecan. Among well-known chemotherapeutic agents causing peripheral neuropathy [34], 4.1% and 0.7% of patients used prior cisplatin/carboplatin and paclitaxel/docetaxel, respectively, among total safety population. The interval between these prior chemotherapies and FOLFOX4 treatment had to be appropriate. As a result, patients who were enrolled in J-PMS with complications of any neurologic disorders, including NSAEs, were 1.1% of total safety population, which suggests that the inclusion of these patients in J-PMS did not give a significant influence on the results of this analysis.

Most crucial medications are neuro-protection drugs, and their representative is an infusion of calcium and magnesium, although this intervention still appears to be not conclusive. It is speculated that patients treated with calcium and magnesium were rare, if any, in the six studies. No patients were supposed to be treated with calcium and magnesium in four Western studies, considering the publication year 2004 of the first exploratory result of calcium and magnesium [25] and the period of the four studies. As the study protocol of MASCOT was almost identical to that of FOLFOX4 arm of MOSAIC study and its study period was between August 2004 and December 2006, it is unlikely that an infusion of calcium and magnesium was performed in MASCOT. When the enrollment of J-PMS started in April 2005 in Japan, major concern of Japanese medical doctors were safety and tolerability of oxaliplatin, including neurotoxicity, but not the neuro-protection, as most of them had not experienced the treatment with FOLFOX regimen. Furthermore, Reference [25] had published just before, and its medical significance was not established. As no other better neuro-protection drugs were reported when the six studies were conducted, it is unlikely that the intervention by neuro-protection drugs did affect the results of this analysis.

In conclusion, the cumulative doses of oxaliplatin linked to the onset of grade ≥3 NSAEs were higher in Asian patients than in Western patients. The Asian populations appear to be less susceptible to neurotoxicity of oxaliplatin than the Caucasian populations.
